# Super-Resolution for “Jilin-1” Satellite Video Imagery via a Convolutional Network

**DOI:** 10.3390/s18041194

**Published:** 2018-04-13

**Authors:** Aoran Xiao, Zhongyuan Wang, Lei Wang, Yexian Ren

**Affiliations:** 1 State Key Laboratory of Information Engineering in Surveying, Mapping and Remote Sensing (LIESMARS), Wuhan University, Wuhan 430079, China; xiaoaoran@whu.edu.cn (A.X.); wlei@whu.edu.cn (L.W.); renyexian@qq.com (Y.R.); 2 National Engineering Research Center for Multimedia Software, School of Computer, Wuhan University, Wuhan 430072, China

**Keywords:** super-resolution, video satellite, deep convolutional network

## Abstract

Super-resolution for satellite video attaches much significance to earth observation accuracy, and the special imaging and transmission conditions on the video satellite pose great challenges to this task. The existing deep convolutional neural-network-based methods require pre-processing or post-processing to be adapted to a high-resolution size or pixel format, leading to reduced performance and extra complexity. To this end, this paper proposes a five-layer end-to-end network structure without any pre-processing and post-processing, but imposes a reshape or deconvolution layer at the end of the network to retain the distribution of ground objects within the image. Meanwhile, we formulate a joint loss function by combining the output and high-dimensional features of a non-linear mapping network to precisely learn the desirable mapping relationship between low-resolution images and their high-resolution counterparts. Also, we use satellite video data itself as a training set, which favors consistency between training and testing images and promotes the method’s practicality. Experimental results on “Jilin-1” satellite video imagery show that this method demonstrates a superior performance in terms of both visual effects and measure metrics over competing methods.

## 1. Introduction

A video satellite is a new type of ground observation satellite that has been developed in recent years. Compared with the traditional earth observation satellites, it can “stare” at specific targets for a long time to capture continuous “video” rather than a still “image” of the earth scene, which is thus very suitable for dynamic target monitoring. Super-resolution (SR) technology aims to enhance the resolution of images or videos by reconstructing them with higher pixel densities and richer detail information. It is of great interest in computer-vision-related applications, such as biomedical diagnosis, video surveillance, and remote sensing. Because video satellite imagery takes a higher temporal resolution but a lower spatial resolution than a normal remote-sensing satellite, SR finds direct application in raising its spatial resolution.

### 1.1. Traditional Super-Resolution Algorithms

Traditional super-resolution algorithms can be roughly categorized into MISR (multi-images super-resolution) and SISR (single-image super-resolution), in which SISR can also be classified into diversified methods, such as interpolation, reconstruction, and machine learning. Since super-resolution is a typically ill-posed problem, learning mapping relationships between low-resolution (LR) and high-resolution (HR) images is more promising than ordinary interpolation. In the past several years, SR studies have primarily concentrated on machine-learning-based methods, i.e., example-based learning. These methods build a mapping relation between LR images and the corresponding HR images by learning from a training set and then map LR images in a testing set to HR ones. In 2000, Freeman et al. [1] proposed MRF (Markov random field) to represent the mapping function. MRF is used to process low-level vision tasks considering consistency between adjacent blocks, with the traditional BP (belief propagation) algorithm used for MRF probability inferences. However, this method not only has a huge computational cost but also is heavily subjected to the similarity between the training set and the testing set. Lately, NE (neighbor-embedding) [2] and SCSR (sparse coding) [3] methods have been proposed to promote efficiency and relax the consistency requirement between training and testing samples. NE assumes that HR and LR images share a similar manifold topology and obtains the HR-LR mapping relation by regarding LR image blocks as a linear combination of the nearest neighborhood ones. Junjun Jiang et al. in [4] presented a super-resolution method using smooth regression with a local structure prior (LSP), which assumes that face image patches at the same position share similar local structures, and uses smooth regression to learn the relationship between LR pixels and missing HR pixels of one position patch. SCSR uses the sparsity of the image signal to create a coupled-dictionary of HR and LR images in the training set. After acquiring sparse coefficients of LR testing images by the LR dictionary, an HR image can then be reconstructed with HR dictionary samples and sparse coefficients. Sparse-representation-based approaches are able to achieve competitive performance in a face image super-resolution application but not if the input is corrupted by strong noise. Junjun Jiang et al. [5] proposed a sparse-representation-based method that incorporates smooth priors to enforce similar training patches having similar sparse coding coefficients, which generates superior reconstruction results when the input LR face image is contaminated by large noise. Zhiliang Zhu et al. [6] proposed a faster K-singular value decomposition (SVD) approximation instead of the exact SVD computation, and thus were able to reconstruct an LR image without an external HR training set. Besides this, Lu Tao et al. [7] proposed a unified framework for representation-based face super-resolution through introducing a locality-constrained low-rank representation (LLR) scheme to reveal the intrinsic structures of input images, which improves traditional face super-resolution techniques that treat image noise at the pixel level without considering the underlying image structures. These works have promoted the development of the SR problem significantly. Linwei Yue et al. [8] reviewed this question in detail.

### 1.2. Super-Resolution with a Convolutional Neural Network

With the popularity of deep learning, super-resolution has recently passed a fast development period. In 2014, Dong et al. [9] pioneered a deep convolutional network for image super-resolution (SRCNN), which is an end-to-end reconstructing net. The results indicate that this algorithm achieves the best performance. About two years later, the authors improved the performance of SRCNN by increasing filter size and number of convolutions without changing the depth of the net [10]. Like many other machine learning algorithms, SRCNN downscales training images as LR images by a down-sampling operation (usually bicubic interpolation) at the pre-processing stage. After upscaling to original size in a pre-processing step, these blurry and magnified images are then sent to the network as input. In other words, LR images are not processed directly in this algorithm, which consequently results in increased complexity and computational time cost. To handle this problem, Wenzhe Shi et al. [11] raised an efficient sub-pixel convolutional neural network (ESPCN). This method achieves the image magnification function in the net and thus greatly improves the speed of reconstruction. When a grey image (with size W×H) is processed by the network, the output keeps the same size but with more channels ($W\times H\times c^{2}$, where c is the upscaling factor). Then, a periodic shuffling operation is used to rearrange pixels to produce an enlarged HR image (cW×cH) in post-processing. However, the post-processing that renders a high-dimensional feature layer instead of an image may disrupt the placement of ground objects within the image. In a traditional net structure, the loss function also has a great impact on learning performance. The generally used loss function is mean squared error (MSE), which simply measures differences between a reconstructed image and a ground truth image at the pixel level. Instead of MSE, Feifei Li et al. [12] proposed another type of loss function called “perceptual loss”. They sent output and labels into a pre-trained VGG16 network, which is a classic deep neural network designed by K. Simonyan and A. Zisserman [13], and then calculated the MSE of the relu2_2 feature layer (RELU is an activation function, and relu2_2 represents a specific middle activation layer in the VGG16 network) as the loss function. As hidden layers of a deep learning network represent high-level feature information of images, this loss function improves the ability to capture perceptual differences between the output of the SR network and label images. In practice, although this method reduces the value of the traditional evaluation index, the peak signal-to-noise ratio (PSNR), it does greatly enhance the visual effects of reconstructed images. Besides this, Ledig Christian et al. [14] use a generative adversarial network to create photorealistic images. Haoyu Ren et al. [15] fused several convolutional neural networks together to improve performance. The PixelCNN architecture was employed to define a strong prior over natural images and jointly optimize this prior with a deep conditioning convolutional network in [16], thus resulting in more realistic photos. Weisheng Lai et al. [17] combined the laplacian pyramid with convolutional networks to more quickly solve the super-resolution problem. Bosch Marc et al. [18] designed a GAN-based architecture by densely connected convolutional neural networks (DenseNets) to realize super-resolution for satellite imagery with a factor of up to 8×. For video SR problems, most of the studies exploit temporal characteristics of a video signal (e.g., correlation, consistency, and smoothness) to accomplish super-resolved video frames. Caballero Jose et al. [19] introduced a spatio-temporal sub-pixel convolution network named VESPCN to exploit temporal redundancies and improve reconstruction accuracy. Xin Tao et al. [20] revealed the importance of proper frame alignment and motion compensation for video SR results and proposed a sub-pixel motion compensation (SPMC) layer in a convolutional neural network (CNN) framework to generate visually and quantitatively high-quality results.

### 1.3. Formatting of Mathematical Components

In the era of big remote sensing data, the question of how to efficiently transfer, process, and store massive remote sensing data has become a common concern for researchers [21]. The super-resolution reconstruction technique builds an effective way to obtain high-resolution images from low-resolution images. Because SR relaxes the hardware requirement of remote sensing sensors and the transmission and storage requirements of remote sensing data, it enables applicants to take advantage of high-resolution images at lower costs.

Video satellites have greatly improved the dynamic monitoring capability of satellite remote sensing systems in some typical scenarios, such as resource census, disaster monitoring, ocean surveillance, dynamic target tracking, and dynamic event observation. However, various constraints on the process of satellite imaging happen under a video satellite environment, including but not limited to low spatial resolution due to ultra-long distance imaging, sensor noise, atmospheric disturbance, and image degradation caused by relative motion. Especially, the optical imaging system has to reduce its spatial resolution in the exchange of continuous video, and meanwhile the communication system has to impose heavy data compression to guarantee reliable information transmission under limited sky–earth channel capacity. These factors result in low video quality in terms of spatial resolution and clearness. Therefore, video satellite applications strongly call for techniques, such as super-resolution, to enhance spatial resolution while preserving high temporal resolution.

However, super-resolution for video satellite imagery is confronted with huge challenges due to the inherent characteristics of satellite video. In contrast to normal still satellite imagery (at the sub-meter level), the dynamic imagery captured by a video satellite has inadequate ground spatial resolution (at the meter level). A pixel within a video satellite image may contain a variety of features and the homogeneity of the image block is much weaker. Moreover, since satellite video covers varieties of objects with different spatial scales, homogeneous landform regions with similar textures and appearance structures show statistical diversity in spatial distribution. Therefore, the traditional methods based on a dictionary or example learning are less-well adapted to this diversified image content and activity complexity for precise super-resolution reconstruction.

The recently developed deep convolutional-network-based methods, typically SRCNN [9] and ESPCN [10], can account for diversity in image content and ground object granularity through a large number of training samples. Natively applying them to satellite video super-resolution cannot yield satisfactory results due to the pre-processing adopted by SRCNN and the post-processing imposed by ESPCN. More specifically, the bicubic interpolation in SRCNN results in blurry effects and the vectorization rearrangement in ESPCN affects the spatial relationship of pixels. This paper proposes a five-layer convolutional network with three kinds of output for video satellite super-resolution. Different from SRCNN and ESPCN, our net uses an end-to-end structure without any pre-processing or post-processing, which not only simplifies training and testing procedures but also boosts SR performance for video satellites.

The main contribution of our work can be summarized as follows:

(1) We propose a five-layer end-to-end network structure without any pre-processing and post-processing for the sake of simplicity. As opposed to outputting a high-dimensional feature layer directly and post-processing as in ESPCN, we place a reshape or deconvolution layer at the end of the network to retain the distribution of ground objects within the image.
(2) We employ a different strategy for the loss function: unlike other CNN algorithms that simply calculate loss via output and ground truth images, we create a joint loss by combining output and high-dimensional features of a non-linear mapping network. This operation can take into account layers before and after magnification, which facilitates a more precise mapping relationship between LR and HR images.
(3) In training, we use satellite video data themselves rather than other images to construct training set. This strategy contributes to the consistency between training and testing images in terms of image content statistics, thus enabling the practicality of the algorithm.

In 2015, the Changchun Institute of Optics, Fine Mechanics, and Physics successfully launched the “Jilin-1” video satellite of 1.12 m resolution, which is really a breakthrough for Chinese ground observation satellite systems. In this article, we use these video data for super-resolution by means of the proposed convolutional network. Section 2 particularly presents our method and Section 3 shows experiments and results. The conclusion is drawn in Section 4.

## 2. Methods

For better reconstruction performance in super-resolution applications, we choose video satellite data itself as the training set instead of other image sets. Video frames are more beneficial to the reconstruction of the video itself due to the consistency between training and testing frames in statistical distribution. Thus, our method, by making full use of the video data itself, does not require an extra training image dataset (note that training data and testing data are excluded). In fact, because of the very rich types of ground objects in remote sensing images and different features exhibited at different resolutions, no uniform training dataset for the super-resolution of remote sensing images has been created yet. The use of video satellite data for super-resolution reconstruction can just avoid this problem.

The procedure of our method consists of the following steps. First, we randomly extract a fraction of the video frames from “Jilin-1” video as a training set. Then, we choose images from the rest of the video frames as testing ground truth images (marked as $I^{H}$). Next, we use bicubic interpolation to downscale $I^{H}$ to get the LR image $I^{L}$. The output $I^{SR}$ is aimed to be as close to $I^{H}$ as possible. In this article, the network structure can be divided into several parts as shown below.

### 2.1. Network Structure

Input: SRCNN uses interpolation, such as bicubic interpolation, at the pre-processing stage to upscale LR image $I^{L}$ as the input of the network. To avoid this step and simplify the whole process, we input $I^{L}$ directly into an upscaling network, such as ESPCN. There are two advantages to this idea. First, it can reduce the time spent on the algorithm. Compared to an image obtained by up-sampling, $I^{L}$ has a smaller size, which means that the total number of image blocks with a fixed size sent to the network will be much less. This strategy will definitely shorten the computational time cost. Second, the direct use of $I^{L}$ instead of its up-sampled image as input can readily learn the mapping relationship from LR to HR as the network, which embeds a magnification operation, can build a more complex nonlinear function. In addition, we process true color images instead of grey images or illuminance images, so that when dealing with multispectral (e.g., Multi-Spectral Scanner (MSS) and Thermatical Mapper (TM) images) or hyperspectral images our algorithm remains usable.

Patch Extraction and Representation: Inspired by SRCNN, our network extracts image blocks and represents them at the beginning. We make use of a convolution filter to segment an input image into fix-sized patches (n×n) and transform them into high-dimensional feature vectors.

Non-Linear Mapping: This is the key part of the network, because its ability to express features dominates the algorithm’s performance. The reconstruction of remote sensing data (images or video) has a higher demand for spatial coordinates and radiation compared with natural images; in other words, the reconstructed remote sensing images should be “more clear” in content and be “more accurate” in coordinates for ground objects. Therefore, the entire non-linear mapping net is designed in a shrinkage-expansion manner, where the size of the filter is firstly reduced to pixel level and then grows larger. After reconstructing feature layers, the network achieves pixel-wise prediction for LR image input.

**Figure 1:**
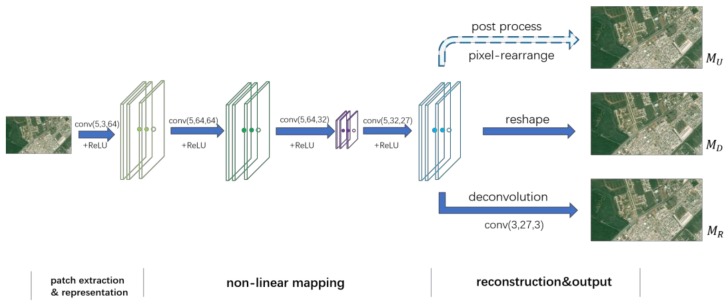
Proposed network structure. “conv(m,n,k) + ReLU” in the figure indicates the input feature maps (or image block at the beginning), which are sent to a convolution layer (m is the kernel size, n and k are the number of layers of feature maps for the input and output, respectively) and an activation layer.

Reconstruction and Output: This part transfers the feature layer of the non-linear mapping network into an HR image. For comparison, we design three ways for reconstruction and output in our algorithm. At first, learning from ESPCN, we output a high-dimensional feature layer directly (marked as $f^{SR}$) to rearrange vectors into a three-channel image in post-processing. We mark this baseline network as $M_{U}$. Particularly, $M_{U}$ learns the mapping function at the pixel level rather than the feature level as its loss function is computed with respect to a high-dimensional vector instead of an image. Alternatively, the second way adds a reshape layer to change the high-dimensional feature layer into three channels (marked as $I_{r}^{SR}$) at the end of the network. Instead of post-processing as in previous methods, $I_{r}^{SR}$ benefits from the net’s simplicity and feature-level learning as well. We label this way as $M_{R}$. Finally, we use a deconvolution layer to take the place of the reshape layer (this output is marked as $I_{d}^{SR}$) and label the network as $M_{D}$. The entire network structure is described in Figure 1.

### 2.2. Loss Function

We take the Mean Squared Error (MSE) to calculate the loss function, which is widely used in the super-resolution problem. There are differences for the three kinds of output. For the first way $M_{U}$, we take the idea of ESPCN and rearrange the pixels of the ground truth image $I^{H}$ by turning it into the same dimensions as $f^{SR}$ and marking these rearranged ground truth vectors as $L_{r}$. Then, the loss function is shown as follows:

(1) $$l_{upscale}=\frac{1}{W\times H\times \left(3\times \mu^{2}\right)}||f^{SR}-L_{r}||{}^{2}.$$

**Figure 2:**
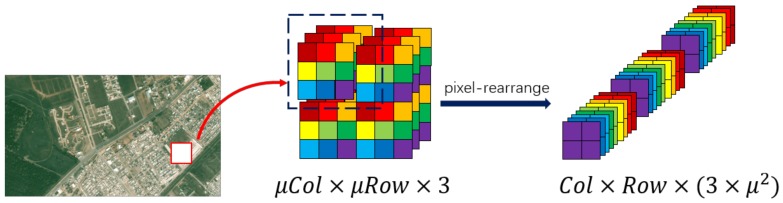
Principle of pixel rearrangement.

For the sake of clear illustration, the used pixel-rearrangement operation is shown in Figure 2.

For the last two ways, we first calculate the output with the ground truth image as follows:

(2) $$l_{R}=\frac{1}{\mu W\times \mu H\times 3}||I_{r}^{SR}-I^{H}||{}^{2}$$

(3) $$l_{D}=\frac{1}{\mu W\times \mu H\times 3}||I_{d}^{SR}-I^{H}||{}^{2}.$$

**Figure 3:**
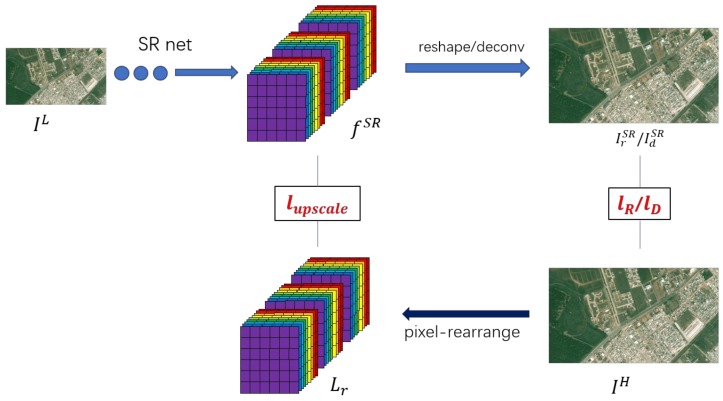
Illustration on loss function. SR = super resolution.

Further, as shown in Figure 3, we combine these losses with the previous $loss_{upscale}$, resulting in the final joint loss:

(4) $$l_{u+R}=w_{upscale}\times l_{upscale}+w_{R}\times l_{R}$$

(5) $$l_{u+D}=w_{upscale}\times l_{upscale}+w_{D}\times l_{D}.$$

### 2.3. Evaluation Index

We choose two commonly used evaluation metrics, PSNR (peak signal-to-noise ratio) and SSIM (structural similarity) to measure the quality of the reconstructed images. They both evaluate differences between a reconstructed image and a ground truth image, but make differences in terms of visual perception.

PSNR, one of the most widely used evaluation indexes in computer vision, is calculated based on the error between the corresponding pixels. It can effectively measure error at the pixel level. However, it may appear that the evaluation results are inconsistent with human subjective feeling as it does not take the visual characteristics of the human eye (for example, human eyes are more sensitive to brightness than chromaticity) into account.

Instead, SSIM measures three aspects of image similarity, including brightness, contrast, and structure. Unlike PSNR, it is based on structural similarity rather than error sensitivity. The larger these two indexes, the better reconstruction we have.

## 3. Experiments and Results

### 3.1. Dataset and Experimental Settings

Both the training and the testing data used in this article were based on “Jilin-1” video satellite imagery. We extracted video frames and selected one for every five frames (so that the total number depends on each video duration) as training data, and we selected test images from the rest at random. We chose several areas in different countries with certain typical types of surface coverage, including vegetation, a water body, and a variety of buildings. Table 1 shows the major properties of experimental imagery.

The convolution sizes of the whole network were respectively set to 5, 5, 1, and 3 with the numbers of filters being 64, 64, 32, and 27. Note that ReLU is appended to each convolutional layer. We compared several common optimizers (including GradientDescentOptimizer, AdamOptimizer, and MomentumOptimizer) for the purpose of faster convergence in training and better performance, and finally decided to take AdamOptimizer [22] with weight decay = 0.98. We set upscaling factor = 3. $w_{upscale}$, $w_{R}$, and $w_{D}$ were set to be 0.5. The patch size was 25. We trained with a batch size of 10 for 100,000 iterations, and the number of epochs was 30. Random numbers in a normal distribution were used to initialize weights. All of the experiments were conducted on a Dell Precision Tower 3620 with NVIDIA Quadro K620 graphics (running in TensorFlow).

### 3.2. Results

We use bicubic interpolation as our baseline and compare our method with SCSR [3] and SRCNN [9] in both visual effect and evaluation index. The former method is a classic traditional machine-learning algorithm, and the latter one is the most widely known CNN algorithm for super-resolution.

**Figure 4:**
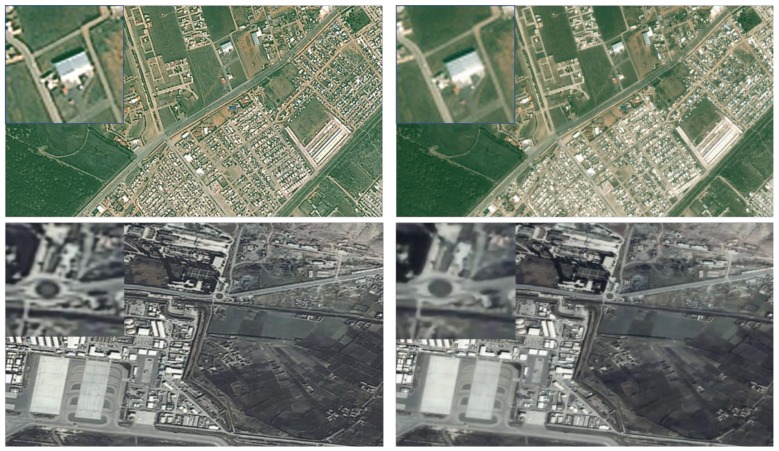
Output of the super-resolution convolutional neural network (SRCNN) using a different training set with “Jilin-1” satellite video (**left**) and Yang91 (**right**). Yang91 is a standard training set that was firstly used by Yang [3].

We notice that the original code published by Dong (running in Caffe) cannot obtain satisfactory results when using video data as training data. As we can see in Figure 4, it (left) produces a similar effect of over-sharpening and results in a higher color distortion compared with output by Yang91 (right), which is a standard natural image set for super-resolution that was firstly used by Yang [3]. This indicates that SRCNN has a limitation for satellite video super-resolution applications.

**Figure 5:**
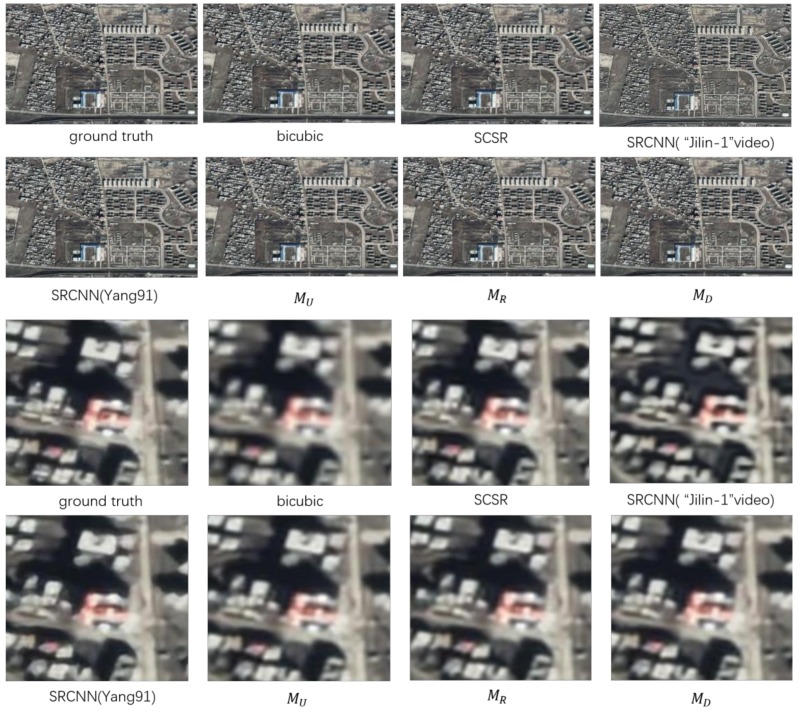
Reconstructed images and details of Kabul in Afghanistan. $M_{U}$, $M_{D}$, and $M_{R}$ represent three kinds of different networks, respectively (see Section 2.1). SCSR = sparse coding.

Figure 5 shows the visual effects of various methods in reconstructing images. All of our three ways can reconstruct the testing image with good visual effects, especially the case with a deconvolution. It confirms that our idea of using satellite video data itself for super-resolution is feasible, which can avoid the extra requirement for a standard training set for remote-sensing video.

From the perspective of quantitative evaluation indexes, our network produces better results compared with competing methods, which are shown as tabulated results in Table 2 and Table 3.

As shown in Table 2 and Table 3, all of our three networks outperform SCSR and SRCNN (in terms of both PSNR and SSIM). Besides, compared with the rearranged pixels in post-processing, the method with a reshape or deconvolutional layer enjoys substantial improvements in terms of PSNR and SSIM. This observation indicates that the end-to-end network can bring us simplicity as well as high quality.

## 4. Conclusions

In this work, we have proposed a super-resolution method using a convolutional network for “Jilin-1” satellite video data. We designed a five-layer convolutional network with three kinds of output, which is trained by a novel joint loss function. We also use satellite video itself as training set to replace other remote sensing training sets, which enhances the practicality of the method. Experimental results on real-world satellite video data show that our method yields a boosted performance in both objective metrics and visual quality.

## Figures and Tables

**Table 1:** Description on experimental videos (from Chang Guang Satellite Technology Co. Ltd.).

Area	Video Duration	Frame Size (Pixels)	Filming Date	Side Swivel Angle
Durango (Mexico)	31 s	1600 × 900	3 February 2016	Unknown
Long Beach (USA)	22 s	3840 × 2160	3 April 2017	3.0424
Tianjin (China)	25 s	3840 × 2160	23 April 2017	21.1707
Kabul (Afghanistan)	15 s	3840 × 2160	23 February 2017	−2.5611

**Table 2:** Peak signal-to-noise ratio (PSNR) of comparison methods. $M_{U}$ , $M_{D}$, and $M_{R}$ represent the three kinds of networks described in Section 2.1. Yang91 is a standard training set that was firstly used by Yang [3].

Testing Images	Bicubic	SCSR	SRCNN “Jilin-1”	SRCNN “Yang91”	$M_{U}$	$M_{D}$	$M_{R}$
Kabul (Afghanistan) (1)	31.88	34.15	26.85	34.03	**35.78**	**36.23**	**35.82**
Kabul (Afghanistan) (2)	34.48	36.70	27.59	36.56	**38.08**	**38.37**	**38.09**
Kabul (Afghanistan) (3)	36.65	38.61	27.76	38.68	**39.60**	**39.88**	**39.67**
Long Beach (USA) (1)	34.92	37.35	29.54	37.38	**38.18**	**39.01**	**38.81**
Long Beach (USA) (2)	37.96	40.83	30.84	40.53	**41.23**	**42.09**	**41.74**
Long Beach (USA) (3)	37.06	39.50	31.33	39.02	**40.09**	**40.82**	**40.58**
Tianjin (China) (1)	34.91	37.15	30.63	36.88	**38.14**	**38.52**	**38.00**
Tianjin (China) (2)	35.57	37.76	31.90	37.34	**38.53**	**38.74**	**38.58**
Durango (Mexico)	31.04	32.83	22.41	32.88	**33.00**	**33.19**	**33.18**

**Table 3:** Structural similarity (SSIM) of comparison methods. $M_{U}$ , $M_{D}$, and $M_{R}$ represent the three kinds of networks described in Section 2.1. Yang91 is a standard training set that was firstly used by Yang [3].

Testing images	Bicubic	SCSR	SRCNN “Jilin-1”	SRCNN “Yang91“	$M_{U}$	$M_{D}$	$M_{R}$
Kabul (Afghanistan) (1)	0.99368	0.99808	0.95539	0.99809	**0.99873**	**0.99888**	**0.99874**
Kabul (Afghanistan) (2)	0.98469	0.99165	0.92958	0.99242	**0.99414**	**0.99465**	**0.99423**
Kabul (Afghanistan) (3)	0.99480	0.99792	0.94538	0.99838	**0.99870**	**0.99884**	**0.99873**
Long Beach (USA) (1)	0.98189	0.99135	0.94770	0.99201	**0.99277**	**0.99449**	**0.99409**
Long Beach (USA) (2)	0.99199	0.99525	0.95648	0.99271	**0.99606**	**0.99725**	**0.99695**
Long Beach (USA) (3)	0.98908	0.99420	0.95111	0.99511	**0.99530**	**0.99634**	**0.99611**
Tianjin (China)(1)	0.98544	0.99839	0.97092	0.99833	**0.99465**	**0.99521**	**0.99452**
Tianjin (China)(2)	0.98735	0.99410	0.96997	0.99416	**0.99543**	**0.99572**	**0.99544**
Durango (Mexico)	0.97333	0.99622	0.86040	0.98767	**0.98273**	**0.98649**	**0.98647**
